# Feasibility of a GATE Monte Carlo platform in a clinical pretreatment QA system for VMAT treatment plans using TrueBeam with an HD120 multileaf collimator

**DOI:** 10.1002/acm2.12718

**Published:** 2019-09-23

**Authors:** Boram Lee, Seonghoon Jeong, Kwangzoo Chung, Myonggeun Yoon, Hee Chul Park, Youngyih Han, Sang Hoon Jung

**Affiliations:** ^1^ Department of Radiation Oncology Samsung Medical Center Seoul Korea; ^2^ Department of Bio‐convergence Engineering Korea University Seoul Korea; ^3^ Department of Radiation Oncology Samsung Medical Center Sungkyunkwan University School of Medicine Seoul Korea; ^4^ Department of Health Sciences and Technology, SAIHST Sungkyunkwan University Seoul Korea

**Keywords:** GATE, HD120, Monte Carlo, TPS QA, TrueBeam

## Abstract

**Purpose:**

To evaluate the quality of patient‐specific complicated treatment plans, including commercialized treatment planning systems (TPS) and commissioned beam data, we developed a process of quality assurance (QA) using a Monte Carlo (MC) platform. Specifically, we constructed an interface system that automatically converts treatment plan and dose matrix data in digital imaging and communications in medicine to an MC dose‐calculation engine. The clinical feasibility of the system was evaluated.

**Materials and Methods:**

A dose‐calculation engine based on GATE v8.1 was embedded in our QA system and in a parallel computing system to significantly reduce the computation time. The QA system automatically converts parameters in volumetric‐modulated arc therapy (VMAT) plans to files for dose calculation using GATE. The system then calculates dose maps. Energies of 6 MV, 10 MV, 6 MV flattening filter free (FFF), and 10 MV FFF from a TrueBeam with HD120 were modeled and commissioned. To evaluate the beam models, percentage depth dose (PDD) values, MC calculation profiles, and measured beam data were compared at various depths (D_max_, 5 cm, 10 cm, and 20 cm), field sizes, and energies. To evaluate the feasibility of the QA system for clinical use, doses measured for clinical VMAT plans using films were compared to dose maps calculated using our MC‐based QA system.

**Results:**

A LINAC QA system was analyzed by PDD and profile according to the secondary collimator and multileaf collimator (MLC). Values for MC calculations and TPS beam data obtained using CC13 ion chamber (IBA Dosimetry, Germany) were consistent within 1.0%. Clinical validation using a gamma index was performed for VMAT treatment plans using a solid water phantom and arbitrary patient data. The gamma evaluation results (with criteria of 3%/3 mm) were 98.1%, 99.1%, 99.2%, and 97.1% for energies of 6 MV, 10 MV, 6 MV FFF, and 10 MV FFF, respectively.

**Conclusions:**

We constructed an MC‐based QA system for evaluating patient treatment plans and evaluated its feasibility in clinical practice. We observed robust agreement between dose calculations from our QA system and measurements for VMAT plans. Our QA system could be useful in other clinical settings, such as small‐field SRS procedures or analyses of secondary cancer risk, for which dose calculations using TPS are difficult to verify.

## 1 INTRODUCTION

Treatment plans of intensity modulated radiotherapy (IMRT) and volumetric modulated arc radiotherapy (VMAT) have dynamic motion of multileaf collimators (MLC), gantry, or dose rate, during dose delivery, and could deliver highly conformal prescribed dose to the target volume while sparing normal volume by modulating intensities.^1^, ^2^ Model‐based dose calculation algorithms in the commercial treatment planning systems such as anisotropic analytical algorithm (AAA) or the collapsed cone convolution class (CCC) have fast dose calculation time and accuracy as level as clinically acceptable.^3^, ^4^

Although accurate beam data measurement and beam modeling can reduce uncertainty of dose calculation in IMRT and VMAT plans, many sources of errors in IMRT planning, including uncertainty of beam modeling, output for small fields, unmeasured out‐of‐field area, heterogeneity, and so on, still remains.^5^ Especially, as VMAT is widely used for stereotactic ablative body radiotherapy (SABR) because of its fast treatment time and high conformality,^6^, ^7^, ^8^, ^9^ importance of the patient‐specific pretreatment quality assurance (QA) has been increased.^5^, ^10^, ^11^, ^12^

Monte Carlo (MC) simulation is a popular method used in comparative studies to verify the accuracy of dose calculation using commercial treatment planning systems (TPSs).^13^, ^14^, ^15^, ^16^, ^17^, ^18^, ^19^ Recently, many methods have been applied to improve the calculation time for Monte Carlo simulation, which is used to verify the accuracy of radiotherapy.^20^ GATE v8.1 which is an open‐source toolkit compatible with the Geant4 medical application system^21^ was released. GATE, which has been mainly used for single photon emission computed tomography (SPECT) and positron emission tomography (PET), is a Geant4‐based MC platform with three‐dimensional simulation and parallel computation.

In the current study, we developed a QA program which can automatically convert treatment plan files in digital imaging and communications in medicine (DICOM) format into our QA system and export three‐dimensional dose in DICOM format for analysis. We used GATE v8.1 as dose calculation engine in the QA system. A TrueBeam with HD120 (Varian Medical Systems, Palo Alto, CA, USA) was modeled and validated as the source of the QA system. To verify the clinical usefulness of the QA program, dose calculations for VMAT treatment plans using the QA program were performed and compared to dose measured using radiochromic film.

## 2 MATERIALS AND METHODS

### 2.1 Design of the QA system

The QA system analyzes plans and converts data into macro files that can be used in GATE. The VMAT plan generated using a TPS exports data to the QA system in DICOM format. The QA system imports plan files and automatically analyzes the treatment parameters, including number of fields, positions of the x and y jaws, positions of the gantry and collimator, and position of the MLC over time for each field and segment. Based on the analyzed information, the QA system creates a folder corresponding to the patient ID and name. It then generates a macro capable of performing dose calculations within the QA system according to number of fields, position of the gantry, field weight, and MLC in the data folder. The MLC is stored with the gantry location information file, with outboard leaf, half‐leaf on target, half‐leaf on isocenter, quarter‐leaf on isocenter, and quarter‐leaf on target divided into parts A and B. The macros (consisting of the time sequences) are split into 200 job players and combined into one file after calculation.

**Figure 1:**
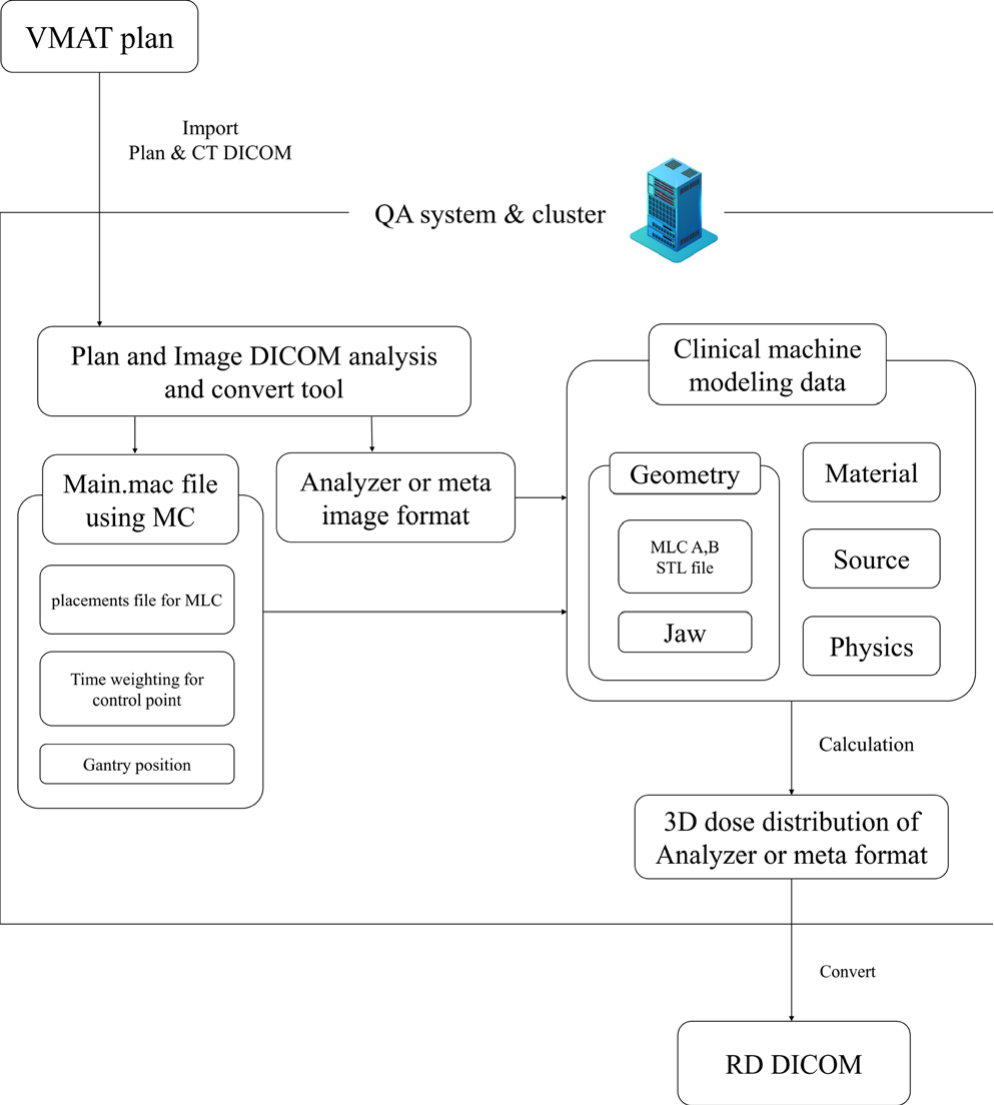
Processing diagram for the Monte Carlo‐based QA system. QA, quality assurance.

To reduce time consumption in MC‐based dose calculation, we designed a compact cluster exclusively. The compact cluster is configured with 88 nodes using the Rocks cluster Linux for dose calculation. The 88 nodes are configured for high‐performance computing (8–16 GB of RAM and a CPU for Intel i7‐3770 and Xeon E3‐1220). The cluster system contains one master node in addition to the calculation nodes. The master node controls the job split, output merge, job submission, and queue management.^21^, ^22^ The master node communicates with the external network to import a treatment plan in DICOM format and perform dose calculation in parallel via a high‐speed ethernet switching hub. The constructed cluster submits and manages the macro converted by the QA system using Condor software.^23^ This QA system was designed to use time weighting applied within the QA program according to the treatment plan. The Fig. 1 shows the configuration and sequence of the system.

### 2.2 Linac modeling

To construct a QA system with Monte Carlo‐based dose calculation engine, geometries of TrueBeam including target, collimator, and HD120 MLC, provided by the manufacturer under non‐disclosure agreement, were modeled^24^ using GATE v 8.1. Modeling of HD120 MLC is the most important for calculating absorbed dose with IMRT/VMAT treatment plan. Geometries from the HD120 MLC were simulated using tessellated volumes from stereolithography (STL) files containing triangular facet data. These data were used to define the surfaces of three‐dimensional objects. It is composed of 32 pieces with a thickness of 2.5 mm at the center and 28 pieces with a thickness of 5.0 mm at the periphery.

Although the detailed geometries of target assembly were modeled, we used phase space files provided by the manufacturer. In the phase space files, photons with nominal energies of 6 MV, 6 MV flattening filter free (FFF), 10 MV, and 10 MV FFF were recorded above the jaws. The photon sources were generated from target and incident electrons with mean energies for each source were 6.18, 5.9, 10.7, and 10.2 MeV, respectively.^22^ The phase space files were formatted according to recommendation of the International Atomic Energy Agency (IAEA) and have been verified in published studies.^21^, ^25^ The average of photon energy is 1.92 MeV and 1.87 MeV for 10 MV and 10 MV FFF, respectively. Because the ratios of photon spectrum with energy over 10 MeV were 0.08% and 0.014%, respectively, hadronic processes was not considered in this study.

In order to validate the modeling of TrueBeam, the three‐dimensional dose in water phantom with size of 60 × 60 × 60 cm^3^ was calculated for each field size varying from 3 × 3 cm^2^ to 40 × 40 cm^2^. Resolution of a voxel was 0.4 × 0.4 × 0.4 cm^3^ and the physics range cut was set to 0.1 mm.^21^, ^26^, ^27^, ^28^ For all the calculations, a source‐to‐surface distance (SSD) of 100 cm was used. The dose calculation results were generated as 3D structures to be analyzed using the program. Each calculated dose was compared with percentage depth dose (PDD) and in‐ and cross‐plane profiles at depth of D_max_, 5 cm, 10 cm, and 20 cm, which were measured with ionization chamber.

### 2.3 Clinical feasibility of the QA system

In order to validate the clinical feasibility, four VMAT plans with photon energies of 6 MV, 10 MV, 6 MV FFF, and 10 MV FFF in stereotactic ablative radiotherapy (SABR) for lung, adrenal gland, liver, and lung cancer, respectively, were enrolled and the characteristics of the plans summarized in Table 1. QA plans, in which the gantry angles of all the constraints in the VMAT plans were set to 0 degree, were generated and exported to the QA system. Three‐dimensional absorbed dose in a 40 × 40 × 10 cm^3^ solid water phantom with a voxel size of 0.2 × 0.2 × 0.2 cm^3^ were calculated using the QA system. The solid water phantom was set up with source to axis distance of 100 cm and depth of 5 cm. The two‐dimensional dose maps at depth of 5 cm were converted to DICOM and compared to the measured absorbed dose using Gafchromic EBT3 film (Ashland, Bridgewater, NJ, USA).

**Table 1:** A summary of treatment plans

	6 MV	10 MV	6 MV FFF	10 MV FFF
Treatment Site	Lung	Adrenal Gland	Liver	Lung
Number of Arcs	2	2	2	2
Number of Control Points	88	182	112	182
Total MU	2429	5319	1781	2730
Daily Dose [Gy]	15	20	12	15

FFF, flattening filter free.

The measured and calculated dose maps were normalized globally to dose that the homogeneously high dose region and nearby prescribed dose were received, following by recommendation of AAPM TG‐218.^5^ The measured dose map was normalized to dose at isocenter of the calculation. The gamma index was evaluated according to the following dose difference (DD)/distance to agreement (DTA) criteria: 2%/2 mm, 3%/3 mm, and 4%/4 mm with 10% dose threshold.

## 3 RESULTS

**Figure 2:**
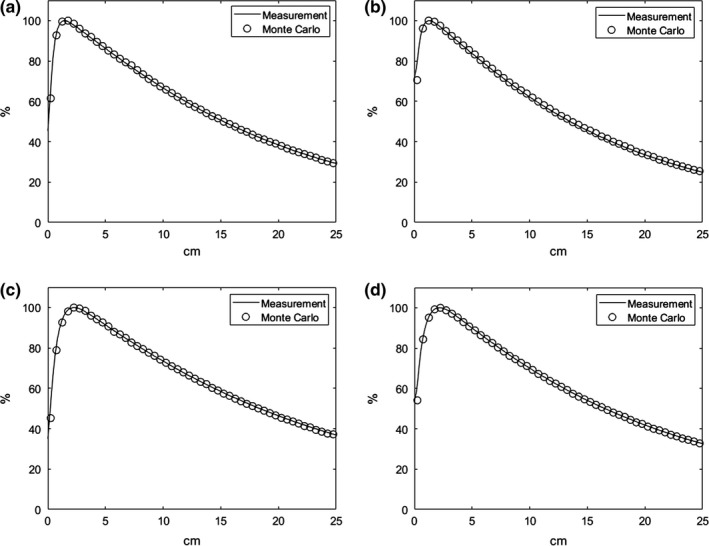
PDD results of a Monte Carlo‐based QA system for (a) 6 MV, (b) 6 MV FFF, (c) 10 M V, and (d) 10 MV FFF energies. The empty circles represent the Monte Carlo calculations, and the solid lines represent the measured data. FFF, flattening filter free; QA, quality assurance; PDD, percentage depth dose.

**Figure 3:**
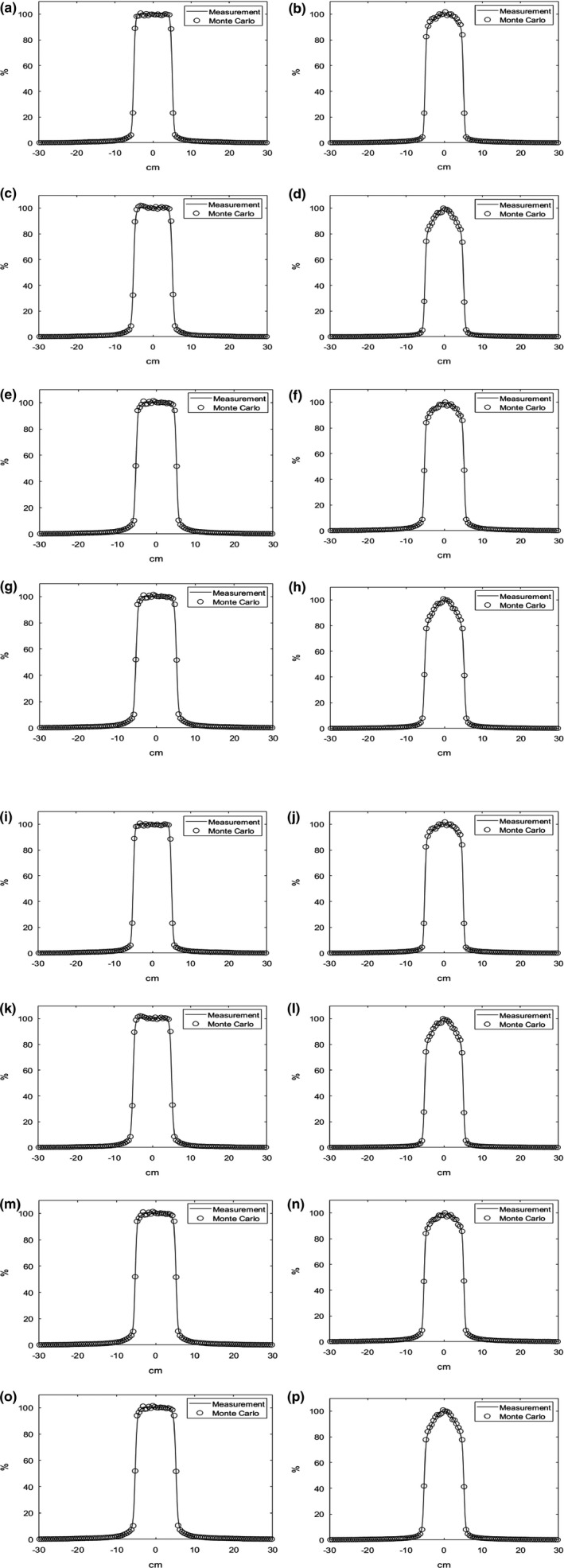
Lateral profile results of a Monte Carlo‐based QA system for (a) 6 MV, (b) 6 MV FFF, (c) 10 MV, and (d) 10 MV FFF energies at Dmax depth, (e) 6MV, (f) 6 MV FFF, (g) 10 MV, and (h) 10 MV FFF energies at 5 cm depth, (i) 6 MV, (j) 6 MV FFF, (k) 10 MV, and (l) 10 MV FFF energies at 10 cm depth, (m) 6 MV, (n) 6MV FFF, (o) 10 MV, and (p) 10 MV FFF energies at 20 cm depth. The empty circles represent the Monte Carlo calculations, and the solid lines represent the measured data. FFF, flattening filter free; QA, quality assurance.

Figure 2 shows the examples of the calculated and measured PDD with the field size of 10 × 10 cm^2^ and Fig. 3 shows the profiles at the depths of D_max_, 5 cm, 10 cm, and 20 cm. The measured and calculated values were represented to be solid line and circle, respectively. The calculated PDD and profiles resulted from MC simulation of TrueBeam seems to be agreed to the measured PDD and profiles well.

Mean errors in PDD and profiles with the field sizes varying from 3 × 3 cm^2^ to 40 × 40 cm^2^ were summarized in Table 2. Each PDD and profile was normalized to D_max_ and central axis dose as 100%, respectively. All the differences at all the measured spots corresponding to the calculated spot were obtained in percentage point and calculated to be the mean errors. The mean errors of the calculated PDDs for all the evaluated field sizes were averaged to be 0.13%, 0.46%, 0.70%, and 0.61% for 6 MV, 10 MV, 6 MV FFF, and 10 MV FFF, respectively. The mean errors of the calculated profiles for 6 MV, 10 MV, 6 MV FFF, and 10 MV FFF were averaged to be 0.45%, 0.38%, 0.44%, and 0.34%, respectively. For all the energies, the smallest average value of the mean errors was observed to be 0.27% for 8 × 8 cm^2^ and largest average value was to be 0.53% for 30 × 30 cm^2^. And, the largest differences between measurements and the Monte Carlo calculation were 1.4% (40 × 40 cm^2^ using 6 MV FFF) and 1.12% (30 × 30 cm^2^ using 10 MV).

**Table 2:** The error rate (%) of the Monte Carlo‐based QA system for (a) PDD and (b–e) lateral profiles

	3 × 3 m^2^	4 × 4 cm^2^	6 × 6 cm^2^	8 × 8 cm^2^	10 × 10 cm^2^	20 × 20 cm^2^	30 × 30 cm^2^	40 × 40 cm^2^
(a) Percent depth dose								
6 MV	0.21	0.03	0.06	0.21	0.12	0.12	0.18	0.11
10 MV	0.32	0.13	0.08	0.32	0.30	0.93	0.54	1.09
6 MV FFF	0.86	0.81	0.89	0.66	0.72	0.73	0.60	0.34
10 MV FFF	0.63	0.84	0.66	0.63	0.76	0.57	0.60	0.23
(b) Lateral profile at D_max_ depth								
6 MV	0.37	0.71	0.15	0.19	0.47	0.88	0.63	0.51
10 MV	0.56	0.60	0.23	0.32	0.21	0.3	0.11	0.37
6 MV FFF	0.05	0.17	0.92	0.78	0.68	0.76	0.70	0.20
10 MV FFF	0.37	0.47	0.26	0.05	0.12	0.61	0.55	0.44
(c) Lateral profile at 5 cm depth								
6 MV	0.66	0.51	0.46	0.49	0.12	0.13	0.84	0.64
10 MV	0.48	0.53	0.62	0.58	0.21	0.31	1.12	0.87
6 MV FFF	0.51	0.42	0.12	0.13	0.29	0.50	0.57	0.52
10 MV FFF	0.55	0.56	0.49	0.43	0.30	0.17	0.09	0.12
(d) Lateral profile at 10 cm depth								
6 MV	0.48	0.70	0.35	0.03	0.61	0.24	0.67	0.51
10 MV	0.84	0.08	0.84	0.34	0.23	0.08	0.01	0.04
6 MV FFF	0.39	0.03	0.15	0.11	0.55	0.51	0.96	0.04
10 MV FFF	0.62	0.46	0.47	0.31	0.17	0.17	0.24	0.11
(e) Lateral profile at 20 cm depth								
6 MV	0.34	0.90	0.57	0.07	0.13	0.48	0.61	0.03
10 MV	0.27	0.61	0.10	0.18	0.42	0.13	0.36	0.32
6 MV FFF	0.48	0.25	0.14	0.24	0.43	0.63	0.57	1.4
10 MV FFF	0.46	0.30	0.47	0.03	0.04	0.21	0.48	0.95

PDD, percentage depth dose.

**Figure 4:**
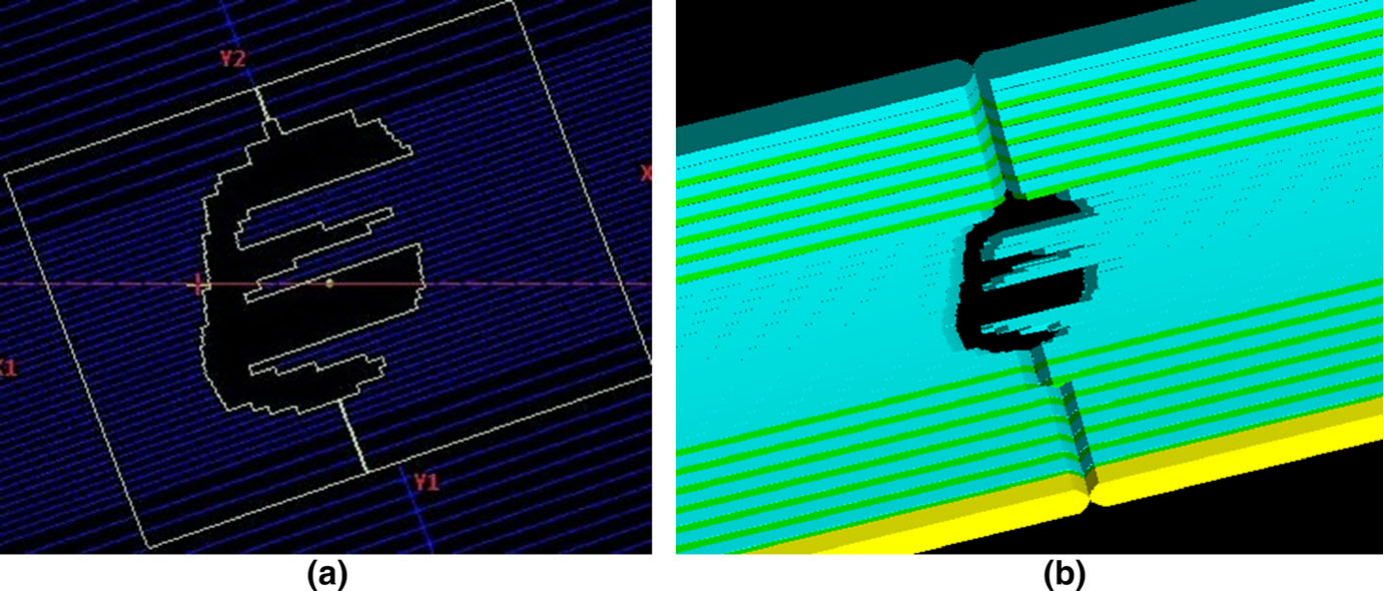
Field shape of the HD120 multileaf collimator generated by (a) a treatment planning system and (b) the Monte Carlo‐based QA system.

**Figure 5:**
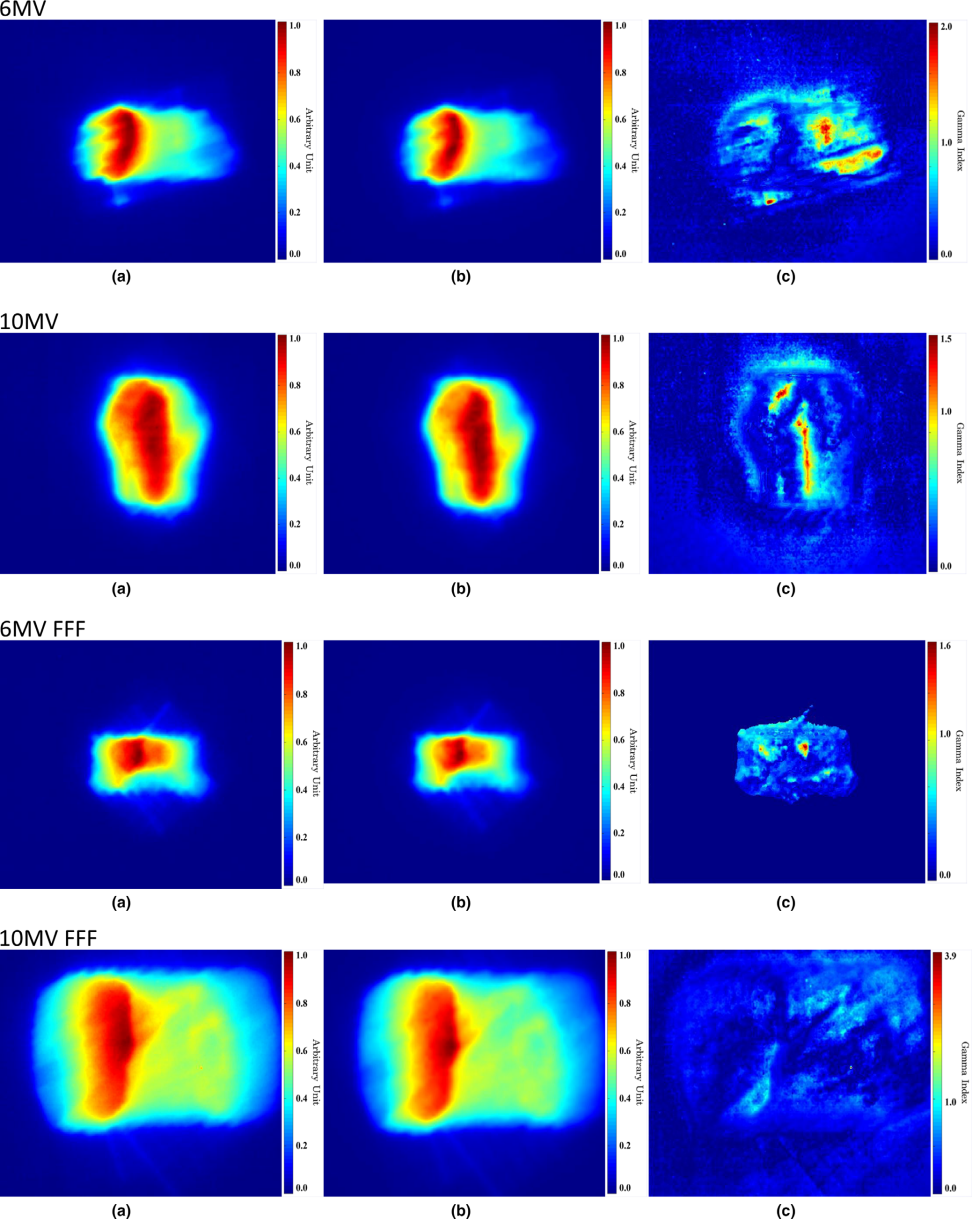
Two‐dimensional dose map for a VMAT plan using four different energies (a) measured by EBT3 film and (b) calculated by the Monte Carlo‐based QA system (SAD 100 cm); (c) the gamma index image. QA, quality assurance; SAD, source to axis distance; VMAT, volumetric‐modulated arc therapy.

The MLC shapes for all the constraints were converted automatically using the QA system, as shown in Fig. 4, and absorbed doses for all the VMAT plans were calculated successfully. Fig. 5 shows the 2D dose distribution from film dosimetry, those from MC‐based calculation using the QA system, and gamma analysis. Gamma values with the criteria of 3%/3 mm were evaluated to be 98.1%, 99.1%, 99.2% and 97.1% in treatment plans with 6 MV for lung cancer, 10 MV for adrenal gland cancer, 6 MV FFF for liver cancer and 10 MV FFF for lung cancer, respectively. Gamma analysis with criteria of 3%/3 mm to 4%/4 mm were evaluated to be over 95% for all the cases, as shown in Table 3. In criteria of 2%/2 mm, gamma value less than 90% was observed for 10 MV FFF.

**Table 3:** Results of gamma index comparison (2%/2 mm, 3%/3 mm, and 4%/4 mm) between measured data using EBT3 film and calculated data using Monte Carlo

	2%/2 mm	3%/3 mm	4%/4 mm
6 MV	94.6%	98.1%	99.5%
10 MV	97.4%	99.1%	99.8%
6 MV FFF	96.5%	99.2%	99.8%
10 MV FFF	87.2%	97.1%	99.8%

FFF, flattening filter free.

The computing time required for MC‐based dose calculation using the QA system with clustering was 15 hours per treatment plan on average. The number of particles and weight for VMAT plans were decided to achieve an overall statistical uncertainty of less than ≤1.0%.

## 4 DISCUSSION

In this study, we constructed a MC‐based QA system with a new version of GATE for the Geant4 platform (released in 2018) and evaluated clinical feasibility. The accuracy of the PDDs and profiles calculated with the developed MC‐based QA system was verified by comparing the measurement results with good agreement of 1%. Especially, the maximum error was 0.92% for the field size of 20 × 20 cm^2^. Dose calculations using the MC‐based QA system for the treatment plans were performed and good agreement with gamma passing rate higher than 97% was observed between the planar dose distributions from the MC‐based QA system and the film measurement. Our results show that MC‐based QA system can be used for the patient specific QA of VMAT treatment plans effectively.

In order to validate feasibility of clinical QA and accuracy in dose calculation, two‐dimensional dose distribution was calculated using the MC‐based QA system and measured using Gafchromic EBT3 film which is one of conventional QA tools for IMRT with high spatial resolution.^29^ AAPM TG‐100 reported various processes involved in the IMRT,^30^ and various sources of uncertainties in the steps of treatment planning and delivery could affect to accuracy of dose calculation and measurement in this study.

In the process of treatment plan, 4‐degree control point in the VMAT treatment plans enrolled in this study might increase uncertainty of delivery.^31^ Inadequate beam measurement, beam modeling, heterogeneity calculation, and region of dose calculation could affect to accuracy of dose calculation.^5^ And also, tissue and material composition could affect accuracy.^32^, ^33^ In order to reduce factor causing dose calculation error, three‐dimensional dose using homogenous solid water phantom was calculated.

In the processes of positioning and delivery, various sources of uncertainties, for example, film positioning, accuracy of leaf motion, and gantry rotation, could affect accuracy of dose delivery. Also, uncertainties of registration and dose conversion^34^ in the step of analysis could affect accuracy of the results in this study. In pretreatment QA for VMAT treatment plan, irradiation with fixed gantry angle and composite dose for the all fields were efficient; however, dose error resulted from uncertainties of MLC motion, gantry rotation, dose rate variation, and daily output variation might be obscured.^5^ Because the purpose of this study was to evaluate accuracy of dose calculation and feasibility of the MC‐based QA system for VMAT treatment plan, we fixed gantry angle to 0 degree in order to reduce and minimize uncertainties of MLC motion and gantry rotation in delivery for VMAT treatment plans.

The purpose of the QA system is to verify dose calculations and other factors related to the actual patient treatment plan. Similar studies have been conducted to evaluate and validate treatment plans.^35^, ^36^, ^37^ Verifying the accuracy of a radiation dose calculation is essential. A reliable, secondary verification should be performed with difficult or complex treatment plans or in stereotactic radiosurgery (SRS) cases requiring high doses in one fraction.^27^, ^38^ Although there are limitations in the dose calculation algorithms used in commercial planning systems, programs for calculating dose using Monte Carlo are often deemed sufficient. However, although Monte Carlo‐based dose calculation has distinct advantages, it can cause differences in results based on the accuracy of position or the material composition^21^ and also it requires more time to calculation. These make the use of Monte Carlo dose calculation difficult in clinical purpose universally.

**Figure 6:**
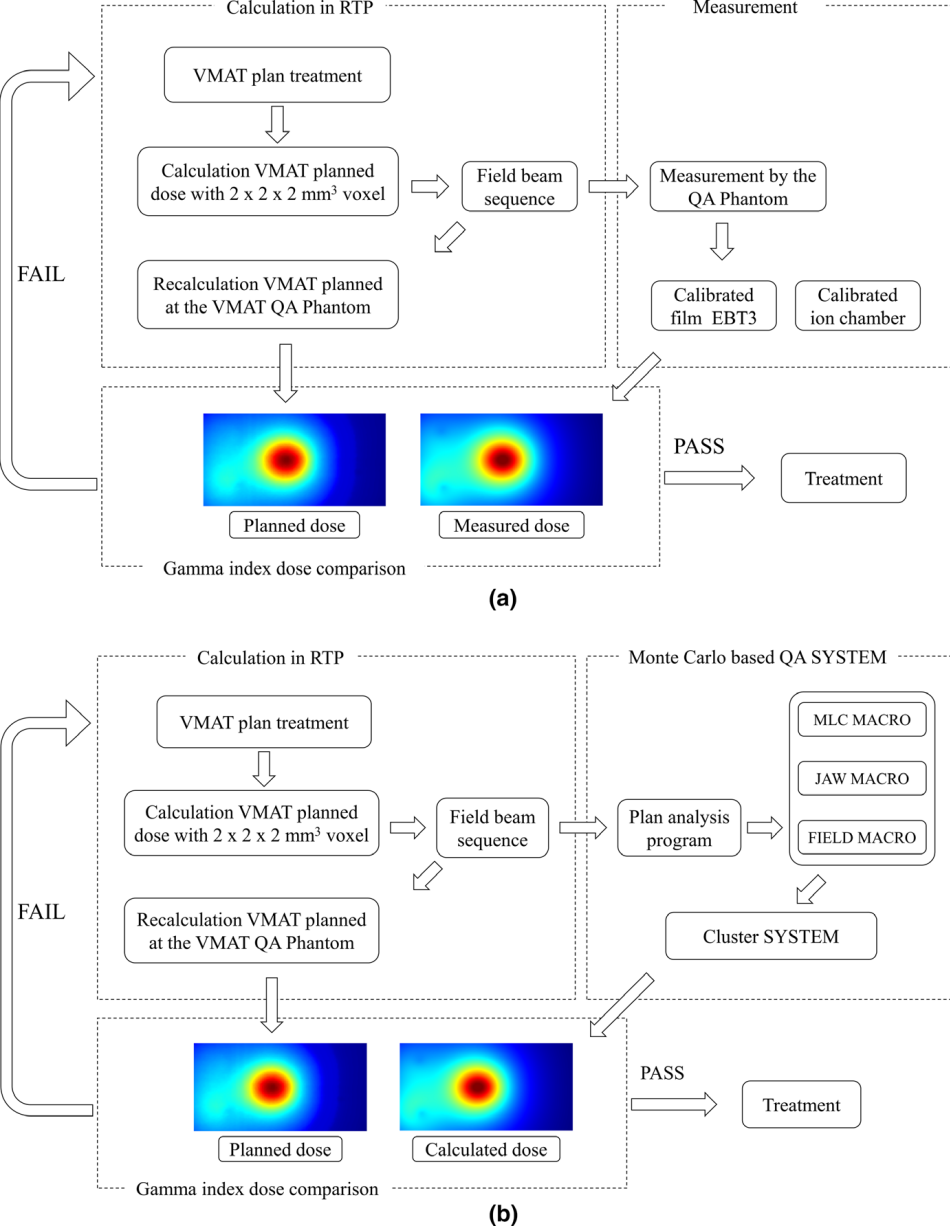
VMAT treatment procedures for (a) measurement‐based QA system currently used and (b) MC‐based QA system. QA, quality assurance; VMAT, volumetric‐modulated arc therapy.

In order to overcome the disadvantages, many researchers are choosing to buy cluster computers at a high cost or to pay for and use parallel computers at sites capable of high‐performance computing. However, it is cost‐prohibitive for many researchers to purchase clusters, and it can be inconvenient to install certain programs or wait for their use. The GATE v8.1 platform used in our study had a problem of compatibility with the existing cluster system, so we upgraded all the programs in the cluster with the QA system. We developed cost‐effective compact clusters that can be constructed directly for each clinical site and developed a QA program that is easily applied and analyzed. The Fig. 6 shows a schematic diagram of the QA program. The current system is a prototype. We are trying to apply SRS or stereotactic body radiotherapy (SBRT) with frequency of about one case per day. We have plans to reduce computation time through future studies. Our Monte Carlo‐based QA system, designed to enable QA before treatment and without the need to access treatment equipment, may be useful for various clinical procedures.

Currently, the QA for VMAT plans used in clinical practice is based on a solid water phantom, which is commonly used to determine the difference from the expected dose in TPS.^39^, ^40^ QA using EPID and dynalog files or independent dose calculations using a treatment plan and dynalog files generated at the pretreatment QA are also being performed. Our developed clinical QA program can identify dose issues that cannot be calculated by TPS, including errors in beam delivery, radiation scattering, and leakage dose. According to the ICRP 86, the incidence of TPS in radiation therapy is 28%. Therefore, the availability of an additional QA protocol system for TPS is becoming increasingly necessary.^41^

As monitor unit (MU) in the use of IMRT or VMAT is usually higher than that in conventional RT, effect of neutron dose in IMRT and VMAT has been studied and reported.^42^, ^43^, ^44^, ^45^ Peripheral dose of thermal neutron in VMAT with 10 MV was less than 100 micro‐Gray of kerma equivalent.^45^ All the mean energies of the photon simulated in this study were less than 10 MeV and only 0.08% for 10 MV was over 10 MeV, we did not consider effect of the neutron dose in film measurement. However, neutron dose generated by the use of photon with higher energy than 10 MV may affect in pretreatment QA and dosimetry in VMAT or IMRT plans.

## 5 CONCLUSION

We established a pretreatment QA system for clinical use of Monte Carlo calculations for TrueBeam and HD120 MLC and confirmed the possibility of using this system for VMAT plans. Our QA system can be used clinically as an additional verification or replacement QA method for VMAT plans, which are increasing. Importantly, our system confirms the accuracy of commercial TPS dose algorithms currently in clinical use. Our system can also be applied in other settings, including calculation of dose accuracy in small fields or secondary cancer risk for out of field which are difficult to assess with TPS.

## CONFLICT OF INTEREST

The authors declare no conflict of interest.
